# Passivating ZnO Surface States by C60 Pyrrolidine Tris-Acid for Hybrid Solar Cells Based on Poly(3-hexylthiophene)/ZnO Nanorod Arrays

**DOI:** 10.3390/polym10010004

**Published:** 2017-12-21

**Authors:** Peng Zhong, Xiaohua Ma, He Xi

**Affiliations:** 1 School of Advanced Materials and Nanotechnology, Xidian University, 266 Xinglong Section of Xifeng Road, Xi’an 710126, Shaanxi, China; pengzhong@xidian.edu.cn (P.Z.); hxi@xidian.edu.cn (H.X.); 2 Key Labof Wide Band-Gap Semiconductor Materials and Devices, Xidian University, Xi’an 710071, Shaanxi, China

**Keywords:** hybrid solar cell, ZnO nanorod array, fullerene modification, surface defect, exciton separation

## Abstract

Construction of ordered electron acceptors is a feasible way to solve the issue of phase separation in polymer solar cells by using vertically-aligned ZnO nanorod arrays (NRAs). However, the inert charge transfer between conducting polymer and ZnO limits the performance enhancement of this type of hybrid solar cells. In this work, a fullerene derivative named C60 pyrrolidine tris-acid is used to modify the interface of ZnO/poly(3-hexylthiophene) (P3HT). Results indicate that the C60 modification passivates the surface defects of ZnO and improves its intrinsic fluorescence. The quenching efficiency of P3HT photoluminescence is enhanced upon C60 functionalization, suggesting a more efficient charge transfer occurs across the modified P3HT/ZnO interface. Furthermore, the fullerene modified hybrid solar cell based on P3HT/ZnO NRAs displays substantially-enhanced performance as compared to the unmodified one and the devices with other modifiers, which is contributed to retarded recombination and enhanced exciton separation as evidenced by electrochemical impedance spectra. Therefore, fullerene passivation is a promising method to ameliorate the connection between conjugated polymers and metal oxides, and is applicable in diverse areas, such as solar cells, transistors, and light-emitting dioxides.

## 1. Introduction

Polymer solar cells have recently attracted extensive attentions in the fields of portable electronics, wearable devices, building-integrated electricity supply and solar-powered airplane, due to their high efficiencies, low cost, light weight, flexibility and compatibility with roll-to-roll manufacturing [1,2,3,4,5,6,7,8,9,10]. Recent studies mainly concentrate on developing new electron donors and acceptors, delicately controlling the nano-morphology and optimizing the device architectures, etc., enabling the efficiencies over 11% [11,12,13,14,15,16]. Upon illumination, excitons generate in the donor of conjugated polymers (sometimes in the acceptor), immediately diffusing towards the donor/acceptor interface, where they are separated into charge carriers. Subsequently, the electrons and holes transport through the n- and p-type semiconductors to be collected by respective electrodes. Generally, high-efficiency polymer photovoltaic (PV) devices rely on three-dimensional interconnected networks of the donor (conducting polymers) and the acceptor (fullerene derivatives), with the sizes of phase separation within the exciton diffusion length (<10 nm) [17,18]. Thus, great endeavors have been committed to optimize the blend morphology of the active layers, leading to complicated processing steps for polymer solar cells [19,20,21,22]. Additionally, employing fullerene derivatives would increase costs [23]. Thus, it is required to find new acceptor materials, which should be cheap, stable, non-toxic, energy-level matching with conjugated polymers and flexibility for controlling their nano-morphology.

ZnO nanorod arrays (NRAs) are regarded as one of the most promising candidates for polymer solar cells [24,25,26,27,28]. They provide a vertically-aligned scaffold prior to deposition of conducting polymers. The sizes of ZnO NRAs (i.e., rod diameter, inter-rod space, and rod length) could be easily adjusted by the hydrothermal reaction conditions [29]. On one hand, the interdigitated structure between ZnO NRAs and conjugated polymers guarantees controllable phase separation. On the other hand, the hybrid ordered bulk heterojunction (BHJ) provides direct channels for charge transport and collection. Additionally, the intrinsic properties of ZnO (e.g., high electron mobility, high electron affinity and large dielectric constant) are all merits needed in polymer solar cells. However, it is reported that ZnO comprises many surface defects, which probably originate from dangling bonds and oxygen vacancies, etc., on the surfaces [30,31]. The defects would introduce additional surface defect states, and thus induce electron trapping and recombination at the donor/ZnO interfaces. This is one of the major reasons for the inefficient exciton splitting and thus the poor performance of ZnO/conjugated polymers based hybrid solar cells. To this end, a series of schemes are designed in order to passivate the surface defects of ZnO [32,33,34,35,36,37,38,39,40,41,42,43]. Chemical doping is a common strategy to tackle the above issue mainly based on two types of dopants, i.e., metal cations (e.g., Bi, Al, Mg) [32,33,34] and anions (e.g., F, N) [35,36]. This method yet suffers from some obvious shortcomings such as: (I) severe morphology change of ZnO after doping, leading to different ZnO/polymer interfaces and twisted percolation pathways for charge carriers; (II) potential lattice distortions for the ZnO matrix accompanying with decreased electron transport; and(III) little effect on eliminating the –OH based defects of ZnO. Additionally, some groups elaborately pre-deposited organo-metallic ZnO precursor into the polymer films, which is subsequently converted into ZnO [37,38]. The insitu formed ZnO/conducting polymer film could substantially improve the interfacial intimacy and reduce recombination. However, it is difficult to obtain highly-crystalline ZnO and precisely control the donor/acceptor nanoscale morphology, as well as remove the oxygen vacancy-based defects by this way. In addition, functionalization of the ZnO surface by a self-assembled monolayer (SAM) or an ultrathin layer of metal oxides seems to be a class of promising approaches to improve interface compatibility, passivate surface states and enhance charge transfer between conjugated polymers and ZnO [39,40,41,42,43,44,45,46,47]. It is essential to select modifiers judiciously, considering the intrinsic nature of ZnO and polymers. For example, phosphonate acid-based SAMs tend to etch ZnO easily due to their strong acid feature [39]. Thiols and silanes are not robust as a SAM, rendering the electronic coupling at polymer/ZnO interfaces unstable [40,41]. Furthermore, the debating conducting polymer/metal oxide interfacial charge transfer is currently not well understood, which calls for continuous studies [48].

In this work, we use ZnO NRAs/poly(3-hexylthiophene) (P3HT) heterojunction as a model system. The nanosizes of the ZnO nanorods can be widely tunable for hybridization with P3HT, so as to facilitate controlling the donor/acceptor morphology at nanoscale. A C60 pyrrolidine tris-acid SAM is delicately selected to modify the ZnO NRAs/P3HT interface. The fullerene buckyball can reduce interfacial incompatibility and enhance exciton disscociations, while the three carboxylic acids of the selected modifier render strong correlations mechanically and electronically between C60 and ZnO. The C60 pyrrolidine tris-acid SAM is expected to passivate the surface defects of ZnO and improve the interface charge transfer and, thus, the performance of PV devices.

## 2. Materials and Methods

### 2.1. Preparation of ZnO NRAs

FTO (SnO_2_:F) substrates (7 Ω/□, Nippon Sheet Glass, Osaka, Japan) were first scrubbed with detergent repeatedly, followed by ultrasonic cleaning in deionized water, acetone, and ethanol for 10 min, respectively. Prior to growth of NRAs, a ZnO seeding layer was deposited onto the FTO substrates by a sol-gel method. Briefly, 0.4 M monoethanolamine and 0.4 M zinc acetate dihydrate was sequentially added into 25 mL 2-methoxyethanol, followed by vigorously stirring at 60 °C in a water bath for 30 min; then the resultant solution was spin-coated onto the FTO substrates at 3000 rpm for 20 s; finally the deposited films on substrates were annealed at 200 °C for 10 min and 500 °C for 1 h, respectively. The thicknesses of the ZnO seeding layer could be controlled by repeating the above steps. The aqueous solution for synthesizing ZnO NRAs comprised 0.025 M zinc nitrate hexahydrate and 0.025 M hexamethylenetetramine. The hydrothermal reactions were conducted by immersing the seeded substrates vertically into the solution at 95 °C for different durations, in order to adjust the nano-sizes of nanorods.

### 2.2. Self-Assembly of C60 Pyrrolidine Tris-Acid

The self-assembly solution was obtained by dissolving 5 mg C60 pyrrolidine tris-acid (Aldrich, Saint Louis, MO, USA) in 100 mL tetrahydrofuran (THF), followed by sonication for several hours to ensure good dispersion. The ZnO/FTO substrates were immersed into the solution for varied time ranging from 0 to 6 h. Then the samples were thoroughly rinsed by THF to remove excess unbounded C60 molecules.

### 2.3. Preparation of P3HT/ZnO Heterojuntions and PV Devices

P3HT (*M*_w_ = 50,000, 95% regioregularity, Merck, Kenilworth, NJ, USA) was first dissolved in chlorobenzene (CB) and stirred at the room temperature overnight. Then the P3HT in CB was deposited onto the ZnO/FTO substrates by spin coating to obtain the P3HT/ZnO heterojunctions. The thicknesses of the polymer films could be controllable by changing the P3HT concentrations and the spin-coating speeds. Immediately, the samples were annealed at 200 °C for 10 min to improve the polymer crystallinity and infiltration. Finally, silver top electrodes with a thickness of about 100 nm were deposited onto the sample by evaporation to complete the devices.

### 2.4. Characterizations

The morphology of the ZnO NRAs was observed by scanning electron microscopy (JSM-7000F, JEOL Inc., Tokyo, Japan). The crystalline properties of the ZnO nanorods were characterized by transmission electron microscopy (TEM, JEOL. JSM-2100F) equipped with selected-area electron diffraction (SAED), and X-ray diffraction (XRD, D/max 2400, Rigaku, Akita, Japan). The infrared spectra were recorded using a BRUKER TENSOR II Fourier transform infrared (FTIR) spectrophotometer (Bruker, Billerica, MA, USA). The UV–Vis spectra and the steady-state photoluminescence (PL) spectra were recorded by a JASCO-570 UV–Vis-NIR spectroscope (Jasco Inc., Easton, MD, USA) and a Hitachi F-7000 fluorescence spectrophotometer (Hitachi, Ltd., Tokyo, Japan), respectively. The dynamic fluorescence spectra were measured in a home-built system by using the scraped (fullerene-modified) ZnO nanorods dispersed in 5 mg·mL^−1^ P3HT solution of CB. The femtosecond laser pulses centered at 800 nm (repetition rate: 1 kHz, pulse width: 65 fs) were employed to excite the fluorescence of P3HT by a two-photon absorption effect. The spectra were recorded by a commercial optical multi-channel analyzer system (OMA, Princeton Instruments, Acton, MA, USA, a gate width of 2 ns), through varying the delay time between the triggering pulse of the OMA system and the exciting pulse. The PV performances of the hybrid solar cells based on P3HT/ZnO NRAs with an active area of 0.07 cm^−2^ were measured by recording the current density-voltage (J-V) curves under an AM 1.5G illumination (100 mW cm^−2^). The incident photon-to-current efficiency (IPCE) was obtained in a QE/IPCE measurement system (Newport, Irvine, CA, USA). The electrochemical impedance spectra (EIS) were conducted witha Zahner Zennium electrochemical workstation (ZAHNER-elektrik GmbH & Co. KG, Kronach, Germany) at a positive bias of 0.3 V in dark, with a magnitude of 10 mV and the frequencies ranging from 10^5^ to 100 Hz.

## 3. Results and Discussion

**Figure 1:**
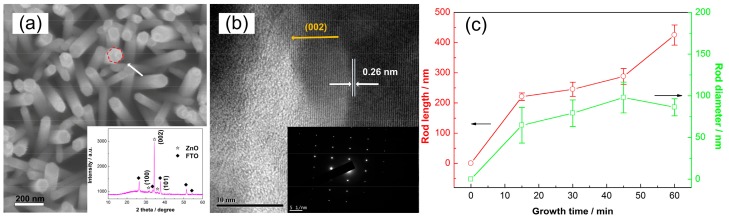
(**a**) SEM top view and XRD pattern (inset) of ZnO NRAs prepared by a hydrothermal reaction; (**b**) high-resolution TEM view and SAED pattern of individual ZnO nanorod; and (**c**) nano-sizes (i.e., rod length and diameter) as a function of the growth durations.

Figure 1a shows the SEM top view of the morphology of the as-grown ZnO NRAs. Uniform nanorod arrays can be obtained through the whole substrate (2.5 cm × 2.5 cm) at present conditions. The tip of each nanorod is observed to be hexagonal as marked in Figure 1a. Additionally, the obtained sparse nanostructures are important for loading P3HT. The XRD pattern as shown in the inset of Figure 1a displays three main peaks, namely (100), (002), and (101), corresponding to the wurtzite phase of ZnO (JCPDS card No. 36-1451). The dominant (002) peak indicates that the nanorods grow preferentially along the c-axis orientation. Figure 1b shows the high-resolution TEM view of individual ZnO nanorod. The lattice fringe with the interplanar spacing is measured to be 0.26 nm, which is assigned to be the (002) lattice plane of ZnO. The SAED pattern as shown in Figure 1b reflects the single crystalline feature of each nanorod. In addition, the nano-morphology (i.e., rod diameter and rod length) of the ZnO NRAs is tunable by controlling the growth durations as shown in Figure 1c, which is flexible for further applications in polymer solar cells. In this work, we choose the sample reacted for 30 min, with a rod diameter of ~80 nm and a rod length of ~250 nm, to prepare solar cells.

**Figure 2:**
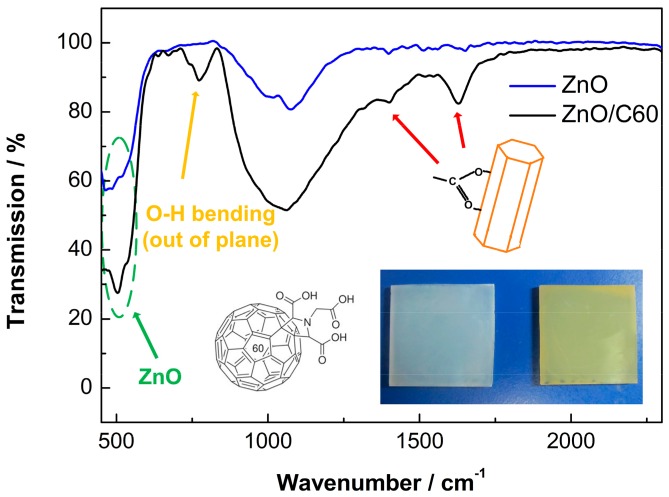
FTIR spectra of the ZnO NRAs with or without graft of C60 pyrrolidine tris-acid; the left inset shows the molecular structure of C60 pyrrolidine tris-acid, and the right inset shows the optical image of the ZnO NRAs before and after modification.

Figure 2 shows the FTIR spectra of the bare and C60 pyrrolidine tris-acid modified ZnO NRA samples. The band located at ~780 cm^−1^ for the modified sample is probably due to –OH bending (out of plane). As shown in the left set of Figure 2, each fullerene molecule has three –OH. It is possible to detect excess ones because not allhydroxyls have the chance to connect with ZnO. The two bands located at ~1420 and ~1600 cm^−1^ might be due to typical carboxylate species, confirming the successful graft of the fullerene onto ZnO [49]. The sample color shows obvious variations after modification as shown in the right inset of Figure 2.

**Figure 3:**
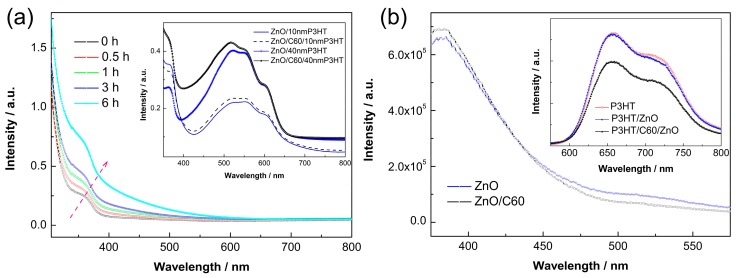
(**a**) UV–Vis spectra of ZnO thin film as a function of the C60 absorption time and the planar structures based on ZnO/P3HT (inset); and (**b**) PL spectra of the ZnO thin films and the planar structure based on ZnO/P3HT (inset).

**Figure 4:**
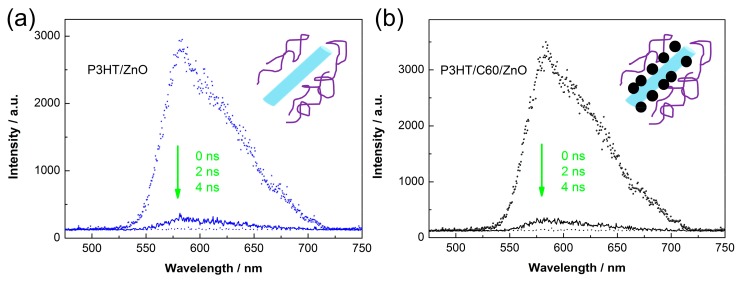
Dynamic fluorescence spectra of the (**a**) unmodified; and (**b**) C60 modified P3HT/ZnO nanorods dispersed in CB. The blue bar, the purple line and the black ball are referred to individual ZnO nanorod, P3HT molecular chain and pyrrolidine tris-acid molecule.

In order to probe theinterfacial properties, simple planar structures are employed based on a 50 nm ZnO thin film prepared by repeating the seeding layers twice. Figure 3a shows the UV–Vis spectra of the C60 modifiedZnO thin films as a function of the self-assembly time. The bare ZnO only shows UV absorption below 380 nm due to its wide band gap (3.2 eV). Prolonging the self-assembly time would cause absorption enhancements in both of the UV and visible ranges (<550 nm), indicating the C60 loading is increased. However, the visible absorption is very small below 3 h. The inset of Figure 3a shows the UV–Vis spectra of the P3HT/ZnO based layered samples with or without C60 SAM for 0.5 h. It can be observed that C60 modification have little influence on the light absorption of P3HT regardless of its thickness. Figure 3b shows the room temperature PL spectra of the ZnO thin films excited at 365 nm. The sharp band centered at ~380 nm corresponds to the inter-band radiation of ZnO (i.e., intrinsic fluoresce). The visible PL might be caused by electron transitions related to defects of ZnO (e.g., oxygen or zinc vacancies, atomic interstitials) [30,31]. It can be observed that fullerene modification apparently improves the intrinsic fluoresce of ZnO, as well as reduces the defect PL. The inset of Figure 3b displays the PL spectra of P3HT planar films. Bare ZnO is observed to be inert to quench the PL of P3HT, suggesting exciton dissociations at the P3HT/ZnO interfaces are inefficient. C60 modification significantly reduces the luminescence, indicating that the fullerene SAM substantially enhances charge transfer between P3HT and ZnO. Based on the above-mentioned results, we believe that the C60 pyrrolidine tris-acid interlayer effectively passivates the surface defects of ZnO and, thus, greatly promotes exciton separations at the donor/acceptor interfaces. Dynamic fluorescence spectra are further carried out to investigate the role of fullerene SAM on charge transport across the P3HT/ZnO nanorod interfaces. As shown in Figure 4, the fluorescence of P3HT completely quenches within 4 ns regardless of a C60 modification. We obtain the integrated areas at 0 ns (*A*_0_) and 2 ns (*A*_2_), and calculate the quenching efficiency (η) within 2 ns by the following equation:

(1) $$\eta =\frac{A_{0}-A_{2}}{A_{0}}$$

As shown in Table 1, the C60 modified sample exhibits larger fluorescence quenching efficiency (0.944) than that of the unmodified one (0.941). The dynamic fluorescence result indicates a more efficient charge transfer occurs at the C60 modified P3HT/ZnO nanorod interface due to passivation of surface defects of ZnO nanorods, which is consistent with the steady-state PL results using layered structures.

**Figure 5:**
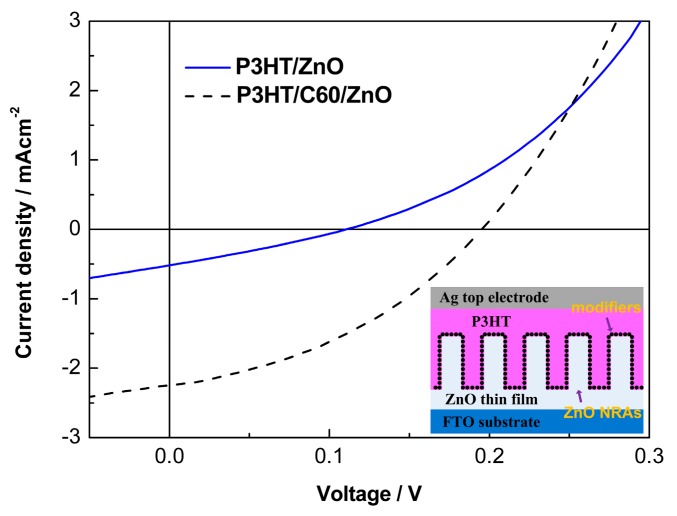
J-V curves of the hybrid solar cells based on P3HT/ZnO NRAs, the inset shows the device structure.

**Figure 6:**
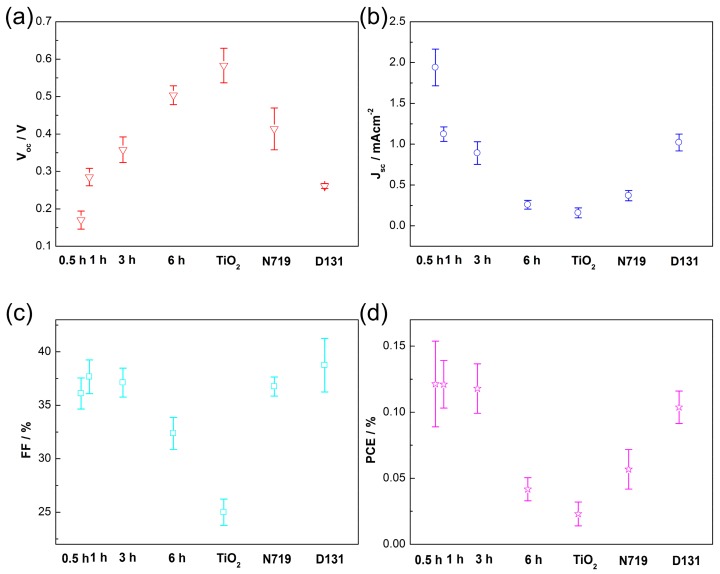
Detailed PV parameters of (**a**) *V*_oc_; (**b**) *J*_sc_; (**c**) FF; and (**d**) PCE as a function of the C60 absorption durations and types of modifiers.

Hybrid solar cells based on P3HT/ZnO NRAs are fabricated with the device architecture as shown in the inset of Figure 5. As shown in Figure 5, the unmodified device show very low PV activity: open-circuit voltage (*V*_oc_) = 0.11 V, short-circuit current density (*J*_sc_) = 0.52 mA cm^−2^, fill factor (FF) = 28%, and power conversion efficiency (PCE) = 0.016%. After C60 modification, the PV performance is improved significantly: *V*_oc_ = 0.20 V, *J*_sc_ = 2.26 mA cm^−2^, FF = 38% and PCE = 0.167%. The introduced fullerene SAM passivates the defects of ZnO NRAs and facilitates exciton separation at the P3HT/ZnO interfaces, leading to the substantially-enhanced device performance. Furthermore, we compare the PV performances of hybrid solar cells with varied C60 self-assembly durations and with other interfacial modifiers. Figure 6 shows the detailed PV parameters of the hybrid solar cells. The V_oc_ continuously increases with the self-assembly time from less than 0.2 V (0.5 h) to ~0.5 V (6 h). As discussed above, more fullerenes loading can be achieved by increasing the immersing time, which better covers the surface of ZnO NRAs, enabling reduced surface defects and recombination. In contrast to *V*_oc_, the *J*_sc_ decreases with the adsorption time consequently from ~2 mA cm^−2^ (0.5 h) to 0.3 mA cm^−2^. This phenomenon might be due to the unfavorable interfacial charge transfer induced by formation of multilayered fullerenes between P3HT and ZnO. Additionally, the FF keeps constant during the first 3 h, and drops at 6 h that is probably due to the functional groups of carboxylic acid tend to etch ZnO. Overall, the PCEs of solar cells are stable below 3 h and displays obvious deterioration at 6 h.

**Figure 7:**
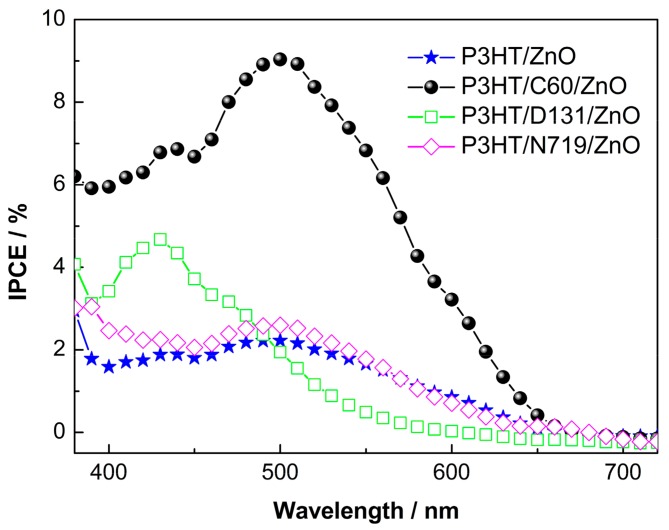
IPCE spectra of the hybrid solar cells.

Some other modifiers are also introduced for comparisons including an ultrathin TiO_2_ interlayer derived by a layer by layer adsorption and reaction (LBLAR) deposition [42], and the organic dyes of N719 and D131. As shown in Figure 6, by using TiO_2_ as interlayer, although a maximum*V*_oc_ of 0.6 V can be attained due to less surface states of TiO_2_ than that of ZnO, the *J*_sc_ and FF are very low, rendering a lowest PCE among all devices. The low electron mobility of TiO_2_ should be responsible. For N719 and D131, it can be observed that their PCEs are inferior to those of C60 modified devices. Figure 7 shows the IPCE spectra of hybrid solar cells. In the 400~650 nm range, the C60 modified device exhibits substantially enhanced IPCE as compared to that of the unmodified one, e.g., from ~2% to over 9% at the peak position of 500 nm, further confirming efficient exciton dissociations at the C60 modified interfaces. While for N719 modified device, only a slightly enhanced IPCE can be observed, indicating that the interfacial charge transfer is inert, considering N719 has overlapped light absorption with P3HT as well. As for the D131 modified solar cell, an extra peak appears centered at ~430 nm contributed by D131 light absorption, while the IPCE due to P3HT absorption from 450 to 650 nm decreases as compared to the unmodified device. This phenomenon suggests the D131 modified solar cell is more likely a dye-sensitized device rather than a hybrid one. Thus, the used C60 pyrrolidine tris-acid is a promising modifier at P3HT/ZnOinterfaces to efficiently split the photo-generated charge carriers by passivation of the surface defects of ZnO.

**Figure 8:**
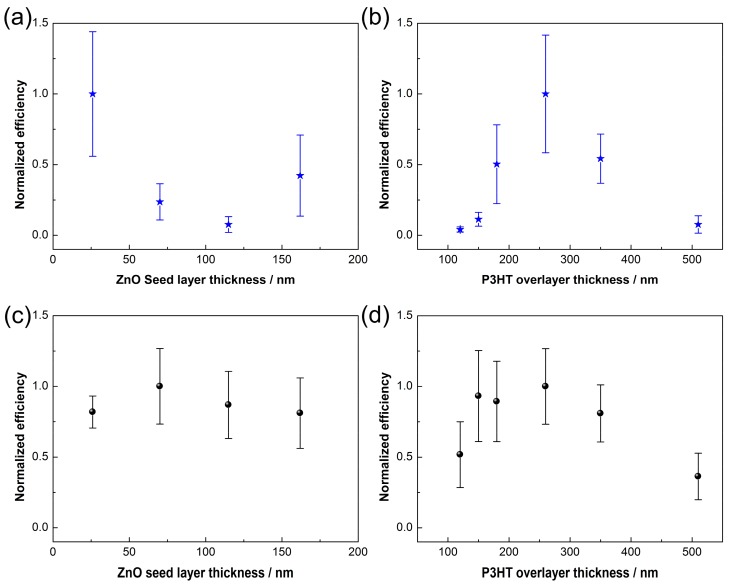
Normalized PCE as a function of the thickness of ETL and HTL for (**a**,**b**) unmodified devices and for (**c**,**d**) C60 modified devices.

**Figure 9:**
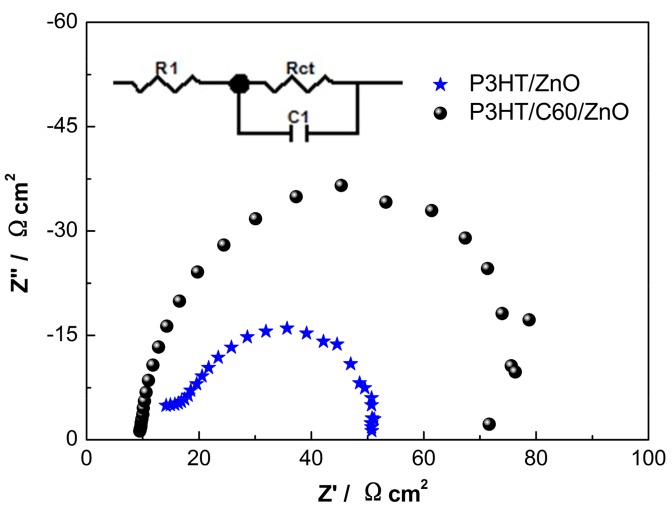
EIS spectra of the hybrid solar cells, inset shows the employed equivalent circuit.

We further optimize the PV devices by studying the effects of thicknesses of the electron transport layer (ETL, i.e., ZnO seeding layer) and the hole transport layer (HTL, i.e., P3HT overlayer), respectively. As shown in Figure 8a,b, without C60 modification, the optimized device performances are attained using a 26 nm ETL and a 260 nm HTL, respectively. The PCE deteriorations with ETL thicknesses might be due to electron scattering at grain boundaries of layer/layer interfaces. The optimized HTL thicknesses is probably attributed to a balance of light absorption and hole transport. Large fluctuations of performance variations are also observed for the unmodified devices. However, after C60 functionalization of the P3HT/ZnO interface, the performance fluctuations of PV devices are more insensitive to the thicknesses of ETL and HTL. This is due to the fact that the C60 modification greatly improves interfacial exciton separations, as well as eliminates the influence of potential pin-holes. An optimized device performance is obtained by using an ETL of 70 nm and a HTL of 260 nm with a fullerene interlayer. Figure 9 shows the EIS Nyquist plots of the hybrid solar cells based on P3HT/ZnO NRAs. After fitted by the equivalent circuit as shown in the inset of Figure 9, the derived parameters related to charge recombination are depicted in Table 2. *R*_1_ is referred to the resistance of the FTO substrate; *R*_ct_ and *C*_1_ are the recombination resistance and chemical capacitance of the P3HT/ZnO NRA interfaces, respectively. The electron lifetime (τ) can be obtained by the following equation [50]:

(2) $$\tau =R_{\mathrm{ct}}\times C_{1}$$

As shown in Table 2, *R*_ct_ is increased from 44.2 to 76.5 Ω·cm^2^, suggesting the C60 SAM serves as a barrier preventing interfacial recombination. Additionally, *C*_1_ is slightly decreased from 2.99 to 2.84 μF·cm^−2^, indicating interfacial charge accumulation is reduced. Furthermore, the τ is obviously prolonged from 91.2 to 135.5 μs. As a result, in our working solar cell, C60 modification passivates defects on the surface of ZnO NRAs, retards back recombination and thus improves exciton dissociations; the vertically-aligned pathways further facilitate the transport of the separated charge carriers, rendering a substantially-enhanced PV performance.

## 4. Conclusions

In summary, vertically-aligned ZnO NRAs with tunable nano-sizes are synthesized by a hydrothermal reaction to be hybridized with the conjugated polymer P3HT. A fullerene derivative called C60 pyrrolidine tris-acid is employed to modify the P3HT/ZnO interface. The C60 modification is observed to suppress the visible fluorescence of ZnO induced by surface defects, and improves the intrinsic PL of ZnO, as well as substantially enhances the charge transfer at the P3HT/ZnO interfaces. C60 modified hybrid solar cells based on P3HT/ZnO NRAs show greatly improved performance and insensitivity with the ETL (HTL) thicknesses, as compared to the unmodified device and the solar cells with other modifiers, which is due to passivation of surface defects of ZnO NRAs, retarded back recombination and enhanced exciton separations. Thus, fullerene passivation should be a versatile method to modify metal oxides and promote its electronic coupling with conducting polymer, which might find widespread applications in hybrid solar cells, photo-transistors, and polymer light-emitting dioxides.

## Figures and Tables

**Table 1:** Quenching efficiencies derived from dynamic fluorescence spectra.

Samples	*A*0	*A*2	η
P3HT/ZnO	209,542	12,294	0.941
P3HT/C60/ZnO	253,133	14,224	0.944

**Table 2:** Parameters related to charge transport and recombination of hybrid solar cells derived from EIS.

Samples	*R*_1_/Ω·cm^2^	*R*_ct_/Ω·cm^2^	*C*_1_/μF·cm^−2^	τ/μs
P3HT/ZnO	12.3	44.2	2.99	91.2
P3HT/C60/ZnO	10.1	76.5	2.84	135.5
